# Correction: Retrograde Ret signaling controls sensory pioneer axon outgrowth

**DOI:** 10.7554/eLife.108945

**Published:** 2025-08-22

**Authors:** Adam Tuttle, Catherine M Drerup, Molly Marra, Hillary McGraw, Alex V Nechiporuk

**Keywords:** Zebrafish

Tuttle A, Drerup CM, Marra M, McGraw H, Nechiporuk AV. 2019. Retrograde Ret signaling controls sensory pioneer axon outgrowth. *eLife* **8**:e46092. doi: 10.7554/eLife.46092.

Published 2 September 2019

It was brought to our attention that one panel in each of Figures 4 and 5 was inadvertently duplicated. We believe these errors occurred during figure assembly in Adobe Illustrator, where some panels were used as placeholders. These errors affect only the specific panels noted below and do not alter the figure captions or any data quantifications.

In the original Figure 4, panel 4I was erroneously duplicated in place of panel 4H. The corrected panel 4H is shown below. Panels 4 H’ and 4I’ displaying the pRet905 channel remain correct.

The corrected Figure 4 (updated for panel H):

**Figure 1:**
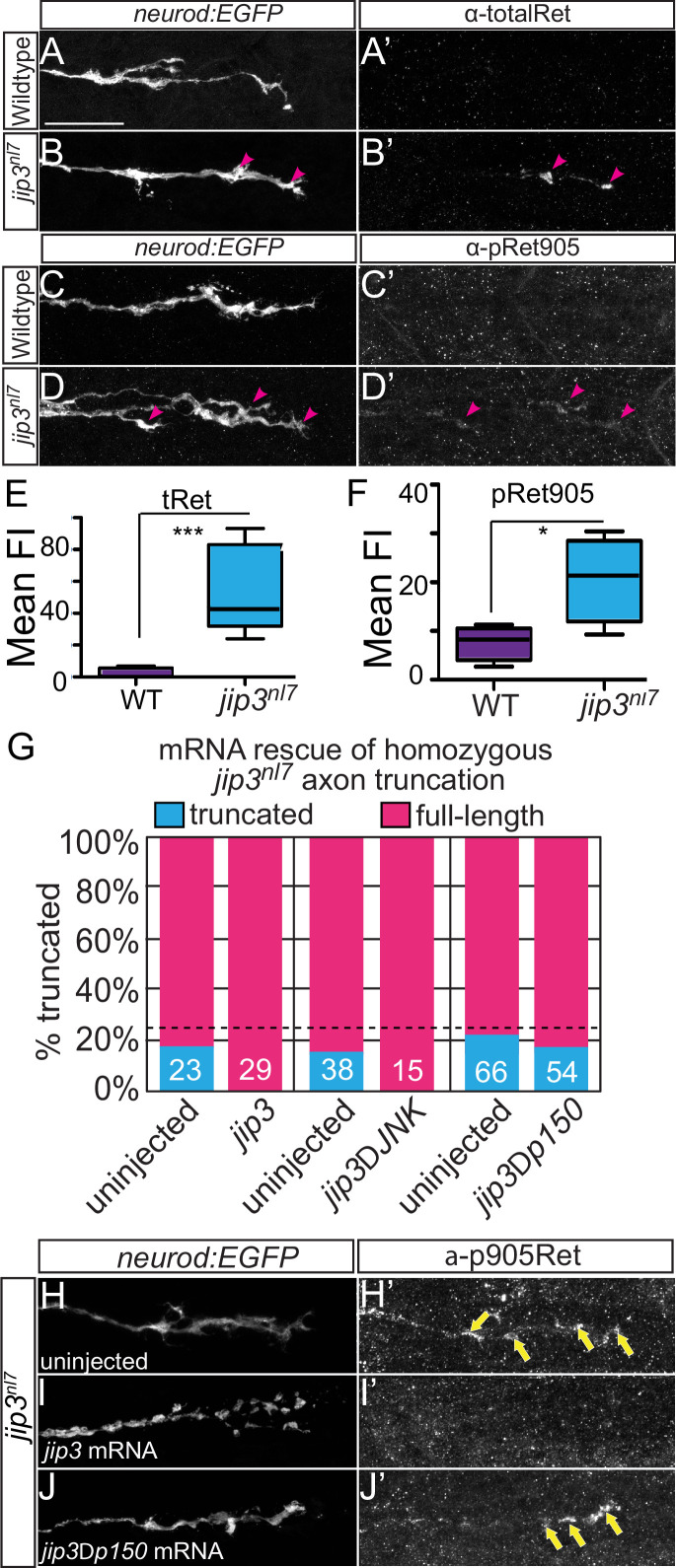


The originally published Figure 4 is shown for reference:

**Figure 2:**
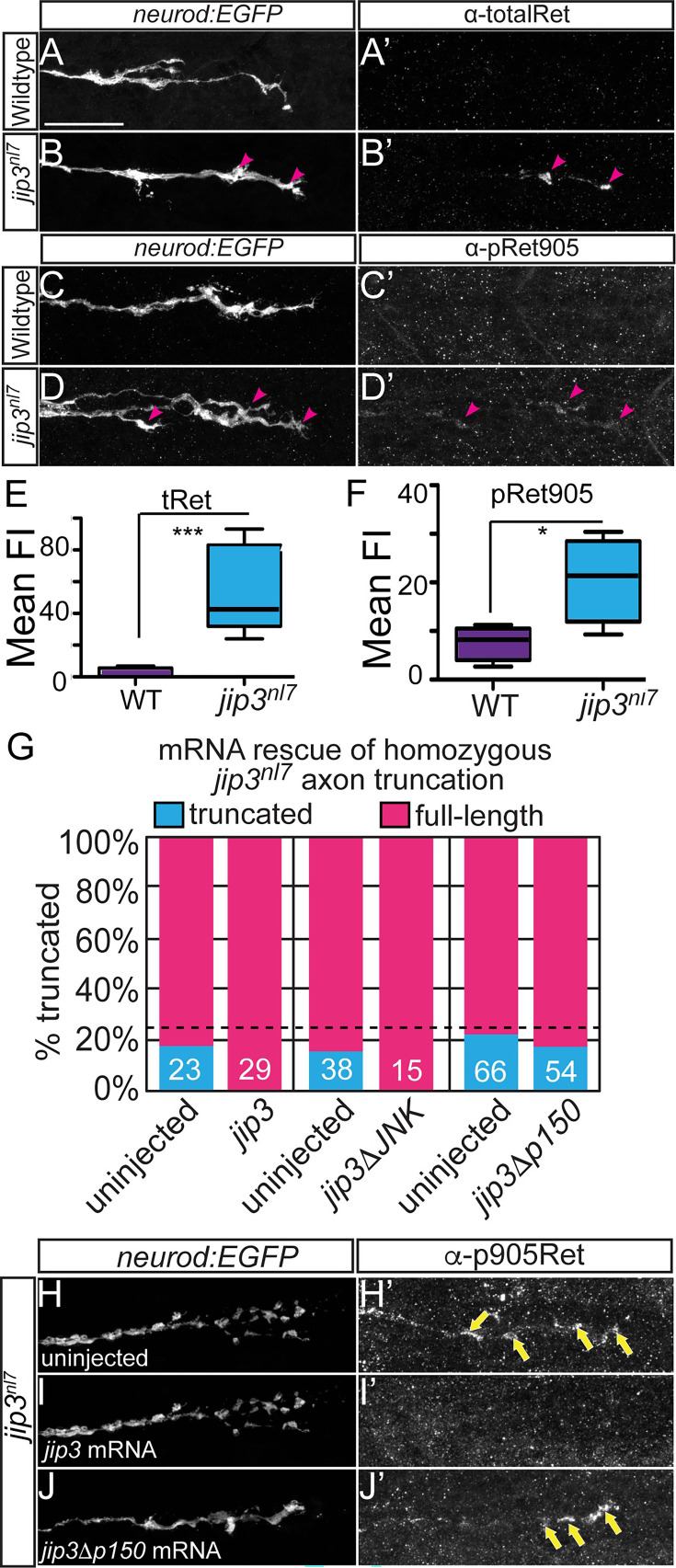


In Figure 5, panel 5C was inadvertently duplicated in place of panel 5D. These images contain two channels: GFP, which outlines the nerve stump, and pRet905 immunolabeling. The GFP channel (not shown in Figure 5) was used to generate the dotted nerve outline for pRet905 quantification. While the pRet905 image in panel 5D was incorrect due to duplication, the GFP channel and thus the nerve outline was correct.

The corrected Figure 5 (updated for panel D) is shown here:

**Figure 3:**
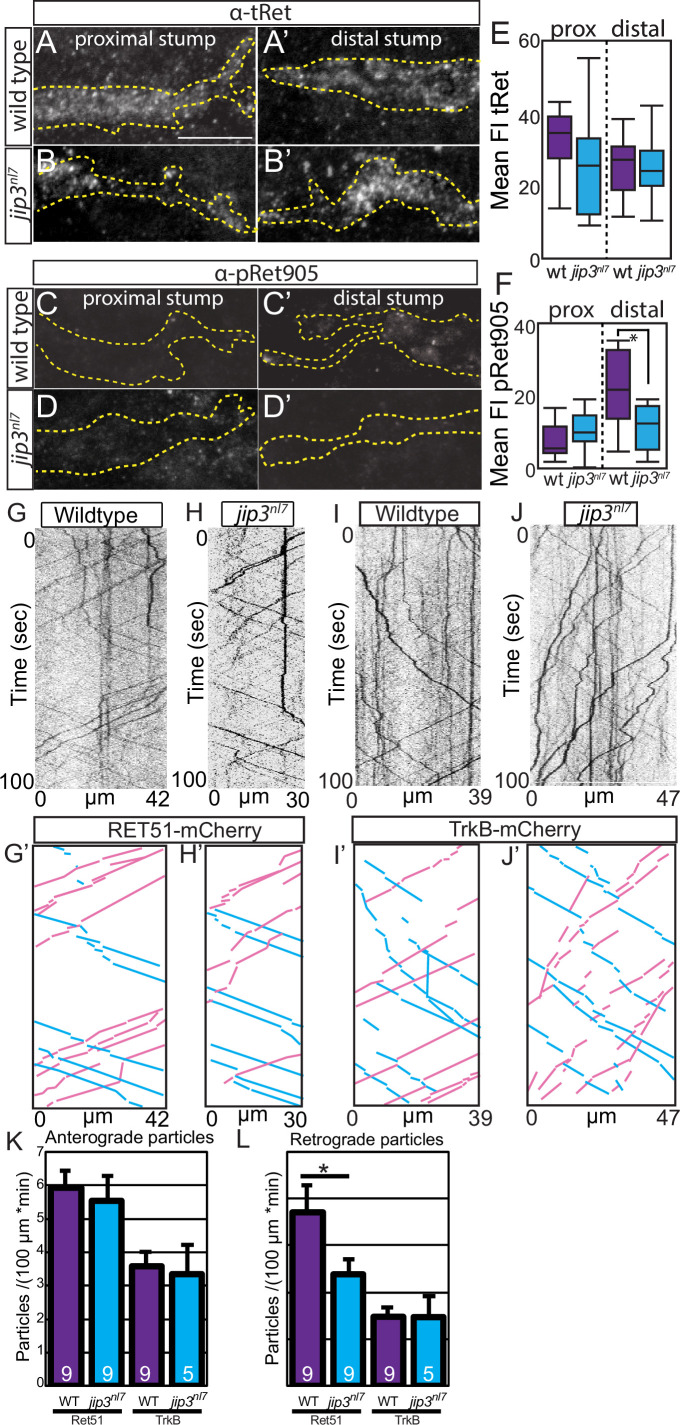


The originally published Figure 5 is shown for reference:

**Figure 4:**
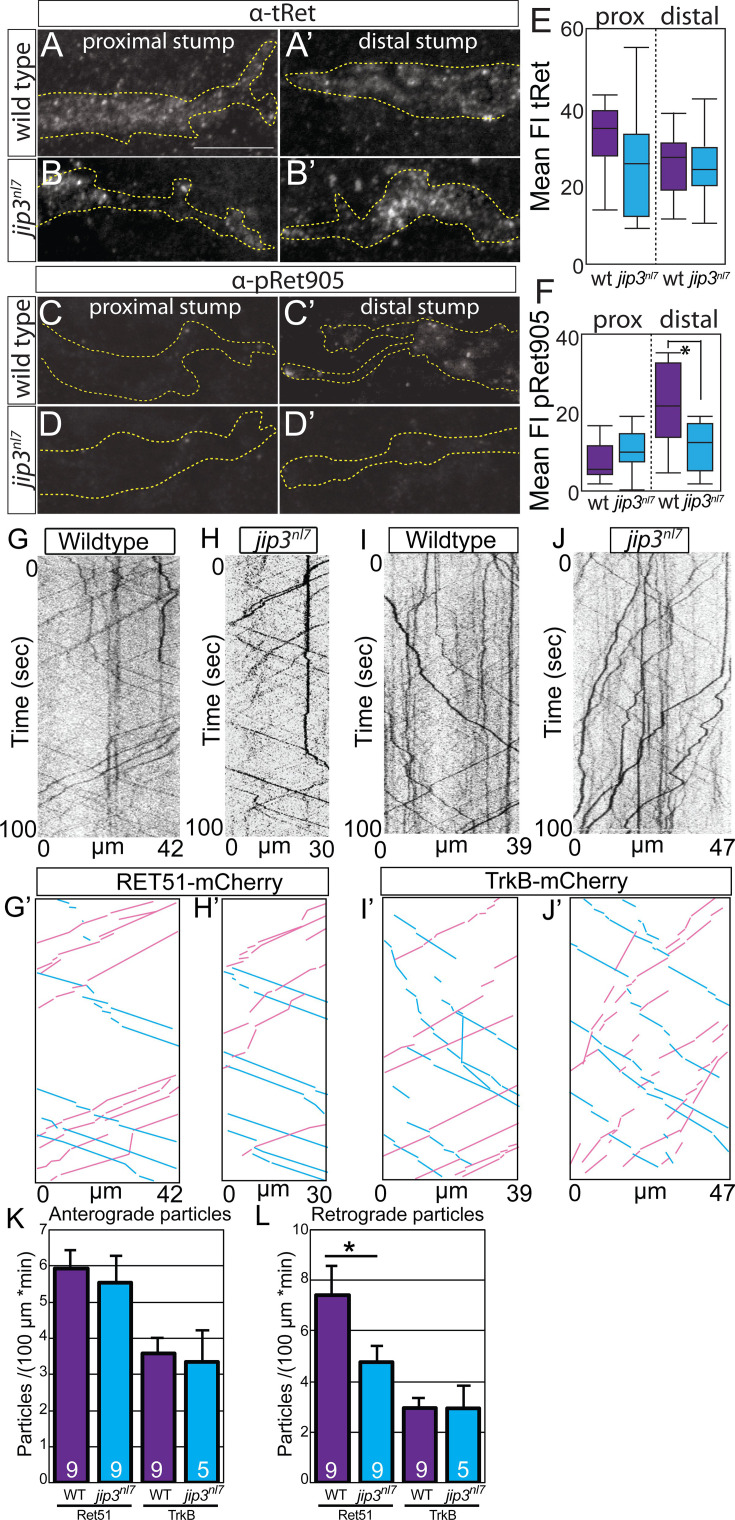


The article has been corrected accordingly.

